# Survivability of Salmonella Typhimurium (ATCC 14208) and Listeria innocua (ATCC 51742) on lignocellulosic materials for paper packaging

**DOI:** 10.1016/j.heliyon.2023.e14122

**Published:** 2023-03-09

**Authors:** Jacob D. Zwilling, Jason Whitham, Franklin Zambrano, Alonzo Pifano, Amy Grunden, Hasan Jameel, Richard Venditti, Ronalds Gonzalez

**Affiliations:** a Department of Forest Biomaterials, North Carolina State University, Biltmore Hall, Campus Box 8005, Raleigh, NC 27695, USA; b Department of Plant and Microbial Biology, 4550A Thomas Hall, Campus Box 7612, North Carolina State University, Raleigh, NC 27695, USA

**Keywords:** Lignocellulosic, Food safety, Paper, Survivability, Bacteria, Packaging

## Abstract

Lignocellulosic materials are widely used for food packaging due to their renewable and biodegradable nature. However, their porous and absorptive properties can lead to the uptake and retention of bacteria during food processing, transportation, and storage, which pose a potential risk for outbreaks of foodborne disease. Thus, it is of great importance to understand how bacteria proliferate and survive on lignocellulosic surfaces. The aim of this research was to compare the growth and survivability of *Salmonella* Typhimurium and *Listeria innocua* on bleached and unbleached paper packaging materials. Two different paper materials were fabricated to simulate linerboard from fully bleached and unbleached market pulps and inoculated with each bacterium at high bacterial loads (10^7^ CFU). The bacteria propagated during the first 48 h of incubation and persisted at very high levels (>10^7^ CFU/cm^2^) for 40 days for all paper and bacterium types. However, the unbleached paper allowed for a greater degree of bacterial growth to occur compared to bleached paper, suspected to be due to the more hydrophobic nature of the unbleached, lignin-containing fibers. Several other considerations may also alter the behavior of bacteria on lignocellulosic materials, such as storage conditions, nutrient availability, and chemical composition of the fibers.

## 1 Introduction

Harmful pathogens can be transmitted through various pathways (e.g., surfaces, direct human interaction, etc.). Consumer behavior has indirectly impacted supply chains due to the SARS-CoV-2 pandemic, leading to surges of nearly 40% in e-commerce sales that heavily rely on the use of corrugated boxes to store, transport, and deliver goods and food products [1]. In 2018, the U.S. generated 33.3 million tons of corrugated boxes, presumably for mainly packaging purposes, while only 14.5 million tons of plastic packaging were generated [2]. In that same year, the recycling rate of corrugated boxes was 96.5% while the recycling rate for plastic packaging was only 13.6% in the U.S [2]. Thus, a large protion of food and other goods are distributed and preserved with lignocellulosic packaging materials and the majority are recycled. As sustainability becomes an increasingly important factor in relation to food packaging, it is likely that paper-based packaging will become even more predominate among consumers and industry.

Foodborne disease caused by bacterial pathogens causes approximately 3.6 million illnesses annually and represent 64% of the foodborne pathogen-related deaths annually in the U.S., with the remaining 36% caused by viruses and parasites [3]. Many of these illnesses, including those caused by *Salmonella* spp. and *L. monocytogenes*, have been linked to human contact with both raw meats and fresh produce, with the transmission emerging from packaging and processing, contaminated irrigation, handler error, improper storage, and contaminated soil systems [[4], [5], [6], [7]]. To the authors’ knowledge, there are no published studies directly relating the number of these illnesses contracted through the direct or indirect contact with paper packaging specifically, although some early reports attribute cross-contamination to be a major route of transmission [8,9]. Nevertheless, it is important to understand the factors that may influence the survivability of foodborne bacterial pathogens on paper-based packaging to guide the development of more advanced technologies for combating foodborne disease.

Several studies have investigated the survivability of common foodborne bacteria on various surfaces and have concluded some general understandings to that regard [[10], [11], [12], [13], [14], [15], [16]]. For example, Siroli et al., 2017, the survivability of *Escherichia coli* and *L. monocytogenes*, among others, on plastic and paper surfaces and found that the plastic surfaces yield higher survivability rates compared to that of paper surfaces. The authors proposed that the sequestration of microbes within the porous fiber matrix of paper materials restricts their access to necessary nurients, whereas such nutrients remain much more accessible on the surface of non-porous plastic materials [11]. On the other hand, Sirsat studied the survivability of *Salmonella* spp., *L. monocytogenes*, and *E. coli* on cardboard coupons revealing that the microbes remain viable for more than 30 days, with the exception of *E. coli*, which maintained viability for only 48 h [14]. However, in the same study, no information was given about the surface material other than it being paper-derived. Investigations of similar microorganisms on stainless steel surfaces have determined that foodborne bacterial pathogens remain viable up to four days on such surfaces [15,16]. In conclusion, the longevity of bacteria on surfaces is highly variable and strain dependent, and access to nutrients seems to be a major contributing factor [[10], [11], [12], [13]].

Paper packaging materials are produced from pulping lignocellulosic biomass containing cellulose, hemicellulose, and lignin. The mass fraction of lignin and hemicellulose present in the paper decreases progressively with the extent of pulping and bleaching processes [17]. In addition to delignification, conventional bleaching methods oxidize the cellulose, resulting in a relatively large number of carbonyl groups [17]. However, the presence of carbonyl groups on the surface of a cellulosic fibers will not likely result in a dramatic increase in antibacterial activity. Lignin, the second most abundant biopolymer present in biomass behind cellulose, has been associated with antimicrobial activity due to its polyphenolic substructure, deeming it a potential candidate for use as a biobased antimicrobial agent [18]. Many studies have investigated the antimicrobial effects of technical lignins extracted from pulp and paper processes. However, more promising results are often found in lignin composites that also contain other antimicrobial agents such as chitosan, cationic polymers, and metal ions [[19], [20], [21], [22]]. Lignin primarily acts as an antimicrobial agent *in vivo* by functioning as a barrier to carbohydrates in plants structures [[23], [24], [25]]. To the authors' knowledge, there has been no investigations comparing the behavior of bacteria on unbleached and bleached paper surfaces. Decoupling the effects of lignin content and oxidation of the cellulosic components during pulp bleaching is conceptually difficult due to the harsh chemical treatment needed to perform such processes and was not investigated in this work.

Herein, we examined the microbial growth and survivability of *Salmonella* Typhimurium and *Listeria innocua* on bleached and unbleached paper substrates. *Salmonella* Typhimurium is a known foodborne pathogen and commonly associated with the majority of Salmonellosis outbreaks [[26], [27], [28]]. *Listeria innocua* has been considered a safe, nonpathogenic surrogate for *Listeria monocytogenes* given their similar biochemical and growth characteristics [29,30]. The paper substrates were made to simulate linerboard for paper packaging applications using fully bleached and unbleached southern softwood kraft pulp at a lignin content relevant to common paper packaging materials (e.g., corrugated paperboard). It was expected that paper-based materials with higher lignin content could inhibit the growth and longevity of bacteria due to lignin's suggested bioactive properties. This study adds to the current literature investigating the survivability of microbes on packaging materials and may serve as a foundation for developing antipathogenic additives for such materials.

## 2 Materials and methods

### 2.1 Materials

Two market wood pulps, southern bleached softwood kraft (SBSK) and southern unbleached softwood kraft (UBSK) pulp from a paper mill in the Southeast United States were used for the formation of paper samples.

Two microbial strains, *Salmonella enterica* serotype Typhimurium MHM 124 (ATCC 14208) and *Listeria innocua* (ATCC 51742), were received as freeze-dried samples from the American Type Culture Collection (ATCC). Trypticase soy broth (TSB) and trypticase soy agar (TSA) were used for overnight cultures incubated at 37°C for both bacterial strains (Fisher Scientific, Waltham, MA, USA). Additional growth factors including dextrose, ammonium iron (III) citrate and anhydrous magnesium sulfate were acquired as well from Fisher Scientific (Waltham, MA, USA).

### 2.2 Fabrication of paper specimens

Paper test specimens were cut from pressed paper handsheets to simulate cardboard with different chemical compositions, specifically lignin content. The procedure for making paper handsheets is described by TAPPI standard methods [31]. The handsheets were made at a basis weight of 120 g/m^2^ and were pressed to simulate a similar structure as linerboard for packaging. These handsheets were set to dry in a humidity-controlled room (23 °C, 50% relative humidity) for 48 h. Once conditioned, the sheets were cut into 2 cm × 2 cm squares. The paper specimens were wrapped in aluminum foil and autoclaved at 121 °C for 60 min before inoculating with the bacteria for sterilization.

### 2.3 Characterization of paper specimens

The paper samples were characterized by their lignin content and associated relative hydrophobicity. The procedure for determining the lignin content of the pulps used for the fabrication of paper handsheets is described by TAPPI T236 cm-85 [32]. The relative hydrophobicity was determined by the water apparent contact angles that were measured by the sessile drop method using a Phoenix 300 contact angle analyzer (Surface Electro-Optics, Suwon City, Korea). Measurements were performed on the first image captured 20 ms after releasing the water droplet to minimize roughness, swelling, and absorptive effects. The water contact angle was estimated using the tangent line-fitting mode from a minimum of seven measurements at various locations on the sheet surface.

### 2.4 Inoculation of microbes on paper

Trypticase soy broth media (TSB) was modified with additional growth factors at the following concentrations: 10 mg/L of ammonium iron (III) citrate and 120 mg/L of anhydrous magnesium sulfate (TSB+). Trypticase soy agar (TSA) media was modified with the same additional growth factors with the addition of 2.5 g/L of dextrose (TSA+). The additional growth factors were added to promote bacterial proliferation in overnight broth cultures and colony formation on agar plates. All broth and agar solutions were autoclaved at 121 °C for 60 min. A solution of dextrose was autoclaved separately then added to the agar solution after sterilization to avoid Maillard reaction byproducts [33].

Each bacterial strain was cultured overnight in TSB+ media at 37 °C. The overnight culture was then transferred to sterile 3 mL vials and frozen overnight at −80 °C. The frozen culture was streaked on the TSA+ plates, then incubated at 37 °C overnight. The colonies from the overnight plates were transferred to a sterile conical tube containing 10 mL of autoclaved saline solution (8.5 g/L of NaCl) and homogenized using a vortex mixer. Bacterial colonies were suspended until an optical density at 600 nm (OD) of 1.0 (∼10^9^ CFU/mL for both microbes) was achieved. Each microbial suspension was diluted by a factor of 10 in 27 mL of TSB + media to make the inoculate suspensions. The paper specimens were inoculated by dropping 0.100 mL of the inoculate suspensions on the paper specimens separately, resulting in a final bacterial load of ∼10^7^ CFU. The paper specimens were inoculated with the bacteria in a broth suspension to simulate a “worst case” scenario, in which the bacteria have optimum conditions to propagate in the initial stages of the experiment [29,34].

Individual paper specimens were allowed to dry for 10 min then placed into 50 mL sterile conical tubes for storage. The samples were stored for up to 40 days at ambient conditions. The conical tubes were opened every other day to allow for air regeneration. For comparative purposes, two alternative storage conditions for the inoculated paper specimens were studied including 1) a dry, high nutrient environment and 2) a dry, low nutrient environment (See supplemental material, Section 1).

The microbes were extracted from the paper specimens by adding 20 mL of a sterile saline solution (8.5 g/L NaCl) to a conical tube with the inoculated paper specimen and agitated using a vortex mixer (MN:12–812, Fisher Scientific, Waltham, MA, USA) at 60% power for 30 s. The resulting suspension was diluted with the sterile saline solution to the appropriate concentration for counting then plated on TSA+ plates. Each condition had 3 replicates for each day of extraction and the entire experiment was repeated for experimental replication (n = 6 per day per condition).

### 2.5 Bacterial enumeration

The bacteria were incubated on TSA+ plates for 24 h for *S.* Typhimurium and 48 h for *L. innocua* at 37 °C. The dilutions were plated over three orders of magnitude to account for major increases or decreases in microbial populations on the paper specimens.

### 2.6 Statistical analysis

All statistical analyses were performed using JMP v. 15 software (SAS, Cary, NC, USA). A test of equal variances (F-test) and student's T-test was performed for determining the significance of differences between the means for each day and paper type. The customized least squares regression fit model was utilized for the comparison of mortality rates. The JSL code for the mortality rate comparisons is provided in the supplementary material (Section 2). The least squares mean comparison was performed using a two-way ANOVA standard least squares regression model. Tukey's HSD multiple comparisons test was used for determining significant differences between groups at a 95% confidence level. Further information on the individual tests is described in later sections.

## 3 Results

### 3.1 Survivability of bacteria on lignocellulosic materials

The two bacterial strains (*L. innocua, S.* Typhimurium) were inoculated on bleached (SBSK) and unbleached (UBSK) paper specimens separately and were stored in ambient conditions over a 40-day period. The concentration of each bacterial inoculum was quantified prior to inoculation of the paper specimens as a control. The number of bacteria extracted from the paper specimens by vortex mixing was not equal for bleached and unbleached samples, thus an extraction efficiency (ε, Eq. (1)) was calculated and used to correct for the actual bacterial populations in each sample for each day studied. The corrected bacterial count, ŷ, was calculated using Eq. (2),

(1) $$\varepsilon ={\bar{y}}_{0}/{\bar{y}}_{c}$$

(2) $$\hat{y}=y_{i}/\varepsilon$$

where ε is the extraction efficiency on the log scale, ȳ_0_ is the average log CFU recovered from paper on Day-0, ȳ_c_ is the average log CFU in the inoculum, and y_*i*_ is log CFU on Day-*i.* The Day-0 point represents the number of bacteria extracted from the sheet 30 min after inoculation. Both papers were characterized by their microbial extraction efficiencies, lignin content, and relative hydrophobicity by contact angle (Table 1).

**Table 1:** Paper characteristics and extraction efficiencies (ε) of microbes from bleached (SBSK) and unbleached (UBSK) pulps. The arithmetic extraction efficiency is the average CFU extracted from each paper type divided by the average CFU in the inoculum.

Paper Characteristics				
Paper Type	Bleached	Unbleached		
Lignin Content (wt.%)	<1^a^	13.0 ± 0.1		
Contact angle (°)	20 ± 3	55 ± 5		
**Extraction Efficiencies**				
Bacteria	*L. innocua*	*L. innocua*	*S.* Typhimurium	*S.* Typhimurium
Paper	Bleached	Unbleached	Bleached	Unbleached
${\bar{y}}_{c}$ (log CFU)	7.3	7.3	7.28	7.28
${\bar{y}}_{0}$ (log CFU)	7.16	6.81	6.86	6.5
ε (log scale)	0.98	0.93	0.94	0.89
ε (arithmetic)	0.77	0.32	0.40	0.17

a The lignin content of fully bleached softwood kraft pulp is below the limit of detection by the kappa test used in this experiment but has been determined elsewhere [35].

The lignin content of the bleached paper was less than 1 wt.%, whereas the unbleached paper contained 13.0 wt.% lignin. Since lignin is a relatively hydrophobic molecule, a higher water contact angle was expected for the unbleached paper (55° vs 20°). The increase in relative hydrophilicity of bleached paper could be due to both the removal of lignin and oxidation of the cellulose during bleaching. The extraction efficiency from the bleached paper was more than twice that of unbleached paper for both microbes, and the extraction efficiency of *L. innocua* was about twice that of *S.* Typhimurium for both paper types (Table 1). The results of the extraction efficiencies show that the bleached samples release more bacteria from the fiber matrix than unbleached samples during vortex mixing. It was observed that the bleached samples lost most of their original structure when vortex mixing (i.e., free suspended fibers), whereas the unbleached samples showed almost no structural change from agitation.

**Fig. 1:**
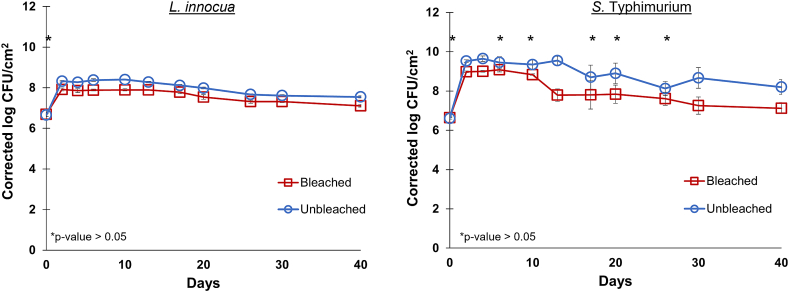
Average log CFU/cm^2^ of *L. innocua* (left) and *S.* Typhimurium (right) on bleached and unbleached paper specimens over a 40-day period. The average log CFU/cm^2^ was corrected for the extraction efficiencies (ε) as described previously. The error bars are representative of the standard error of the mean. Insignificant differences (p-value > 0.05 between the bacterial counts for each day are indicated by an asterisk.

The number of bacteria extracted from the bleached and unbleached paper specimens for each of the microbes was studied over a 40-day period under a worst-case condition (moist, nutrient-dense environment) (Fig. 1). Both bacteria species seem to not only survive but proliferate ∼500-fold after 48 h on the paper specimens due to the nutrients present in the inoculum (Fig. 1). After 40 days, the viable microbial populations are still higher than their initial population extracted 30 min after inoculation. The growth and survivability of the bacteria was also tested over a three-day period under less favorable conditions, specifically a drier and less nutrient-enriched environment (Supplementary Section 1). Under drier conditions but a nutrient-dense inoculum, the bacterial growth was shortened by one day compared to the worst-case scenario and a reduction of the overall population to the level of the initial inoculum within three days (Fig. S1). In contrast, in the absence of excess nutrients (saline inoculation media) and continuous air regeneration, the bacteria were at populations lower than the limit of detection after 24 h (Fig. S1).

**Fig. 2:**
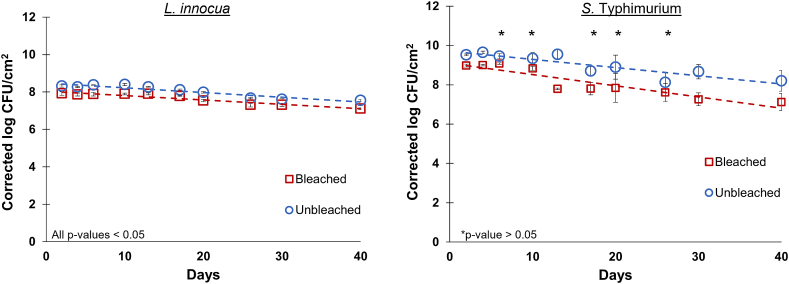
Standard least squares regressions for *L. innocua* (left) and *S.* Typhimurium (right) on bleached and unbleached paper specimens over a 40-day period. The average log CFU/m^2^ was corrected for the extraction efficiencies (ε) as described previously. The error bars are representative of the standard error of the mean. Slopes, y-intercepts, and R^2^ values are displayed in Table 2. Insignificant differences (p-value > 0.05 between the bacterial counts for each day are indicated by an asterisk.

In consideration of the extensive growth of the bacteria in the first 48 h, followed by a gradual decline thereafter, a non-linear cell growth and decay model was performed on the dataset from day 0 to day 40 (Fig. S2, Table S1). This non-linear model fits reasonably well, but the growth parameter is not very accurate due to the limited number of data points taken within the growth stage. Given that the dataset was corrected for the extraction efficiencies and the inoculum concentrations were roughly the same (Table 1), the Day-0 data points are therefore trivial for all conditions, and a more representative linear model was applied to the dataset from days 2 through 40 (Fig. 2).

From the linear regressions, the mortality rates (β) are reasonably similar between all four conditions with greater variability present in the *S.* Typhimurium samples (Table 2). It should be noted that the *S.* Typhimurium samples exhibited much greater variability between replicates than the *L. innocua* samples, thus explaining the relatively low R^2^ -values (Table 2). However, the variability reported in this work is comparable to previously reported studies where similar microbial species were examined [[13], [14], [15], [16]]. To test the statistical significance of the differences between slopes, a customized least squares comparison model was performed on the dataset from days 2 through 40 (Table 2). The JSL code for this custom test is supplied in the supplementary material (Section 2). From the data analysis, there is enough evidence to suggest with 95% confidence that *S.* Typhimurium on bleached paper decays significantly faster than the *L. innocua* samples on either paper type. The samples containing *S.* Typhimurium exhibited considerably higher growth after 48 h of incubation (ȳ_max_) compared to the samples containing *L. innocua* (Fig. 2, Table 2).

**Table 2:** Fitting parameters for each of the linear regressions displayed in Fig. 2. Values for the parameters were calculated using JMP standard least squares fit model with paper and bacteria as fixed effects as well as the interaction term. The comparison of slopes test was computed using a customized least squares regression (see supplementary material, Section 2). A p-value <0.05 is considered a statistically significant difference.

Parameter	Bleached	Unbleached	
*L. innocua*			
ȳ_0_	6.686	6.655	
ȳ_max_	8.064	8.493	$y={\bar{y}}_{\max}+\beta t$
Mortality (β)	−0.024	−0.026	
R^2^	0.73	0.82	
*S.* Typhimurium			
ȳ_0_	6.643	6.607	
ȳ_max_	9.092	9.708	$y={\bar{y}}_{\max}+\beta t$
Mortality (β)	−0.057	−0.042	
R^2^	0.39	0.24	

*Comparison of Slopes (α = 0.05)*					
Bacteria	Paper	-Bacteria	-Paper	β1-β2	P-value
*L. innocua*	Bleached	*L. innocua*	Unbleached	0.002	0.881
*L. innocua*	Bleached	*S.* Typhimurium	Bleached	0.033	0.002
*L. innocua*	Unbleached	*S.* Typhimurium	Unbleached	0.016	0.129
*S.* Typhimurium	Bleached	*S.* Typhimurium	Unbleached	−0.016	0.120

**Fig. 3:**
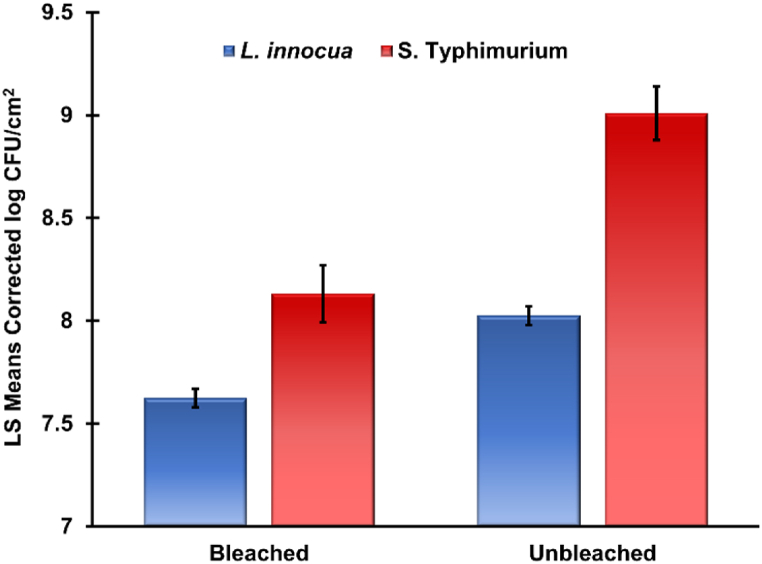
Least squares (LS) means of each of the four conditions studied. Error bars are representative of the 95% confidence interval.

In contrast to the initial hypothesis, the lignin-containing unbleached paper samples yielded higher viable bacterial populations for each day studied, for both types of bacteria (See Figs. 1 and 2, Table 2). To confirm the significance of these effects (bacteria, paper), a statistical comparison was performed on the dataset (Table 3). The time interval of interest is after the initial growth period between days 2 and 40 for reasons previously stated. Therefore, the Day-0 data points for all paper types and bacteria were omitted. A least squares means plot is included in the analysis for graphical representation of the differences (Fig. 3).

**Table 3:** Comparison of least squares means for each of the four conditions tested. A p-value <0.05 is considered a statistically significant difference.

Paper	LS Mean (μ)	Std. Error
*L. innocua*		
Bleached	7.62	0.045
Unbleached	8.03	0.045
*S. Typhimurium*		
Bleached	8.13	0.139
Unbleached	9.01	0.130

*Tukey HSD Comparison of Means (α = 0.05)*					
Bacteria	Paper	-Bacteria	-Paper	μ1-μ2	P-value
*L. innocua*	Bleached	*L. innocua*	Unbleached	−0.402	0.007
*L. innocua*	Bleached	*S.* Typhimurium	Bleached	−0.455	0.001
*L. innocua*	Unbleached	*S.* Typhimurium	Unbleached	−0.941	<0.001
*S.* Typhimurium	Bleached	*S.* Typhimurium	Unbleached	−0.888	<0.001

The results from Table 3 and Fig. 3 suggest with 95% confidence that unbleached paper yields higher viable bacterial populations than bleached paper for both microbial species studied. When combining these results with the comparison of slopes in Table 2 and Fig. 2, a few conclusions can be made. The microbial growth in the first 48 h is higher for *S.* Typhimurium on paper surfaces than *L. innocua*. The mortality rates are statistically similar between groups apart from *S.* Typhimurium on bleached paper which decays at a slightly higher rate than the *L*. *innocua* bleached and unbleached samples. There was no significant difference of mortality rates between the different paper types for *S.* Typhimurium*.*

## 4 Discussion

The aim of this study was to determine if there was a difference in bacterial growth and survivability of bleached and unbleached paper surfaces for both *L. innocua* and *S.* Typhimurium bacterial strains. It was determined that paper substrates containing a higher lignin content (unbleached) allowed for higher levels of microbial growth compared to bleached paper with minor differences in mortality rates over a 40-day period. This conclusion contradicts the initial hypothesis that lignin in its lignocellulosic form would have a negative effect on bacterial growth and survivability. The difference in chemical compositions of the two paper materials is more complex than lignin content, such as oxidation of cellulose and hemicellulose during bleaching. Decoupling these components is conceptually difficult given that delignification involves some degree of oxidation of cellulose, such as the case for fully bleached market pulp [17]. This presents a limitation in this study regarding the root causes of the bacterial growth and survivability differences on the two surfaces. Future work could address this limitation by controlling pulping and bleaching conditions and tracking the growth and survivability of bacteria on the resulting materials. Nevertheless, the significance of this work is apparent in identifying the high potential for bacterial contamination and viability on industrially significant paper materials developed for packaging.

One potential reason for unbleached paper favoring microbial growth could be the more hydrophobic nature of the fibers derived from the higher lignin content, as evidenced by a greater water contact angle. Zambrano et al. determined that unbleached pulp has a higher absorption capacity for water than bleached pulp due to the increased interstitial spaces between fibers (supplementary material, Fig. S3), whereas the bleached pulp retains much more water within the fiber cell wall, as evidenced by a higher wicking rate [17]. The more hydrophobic nature of the fibers may reduce the amount of wicking of broth media from the inoculum, resulting in more nutrients available to the bacteria in the form of droplets in the interstitial space between fibers during the early growth stages. This would explain why the unbleached paper had consistently higher microbial growth after 48 h of incubation. Since the mortality rates of the microbes were similar, the unbleached paper will have consistently higher bacterial populations since they grew to a much greater number initially. If the inhibition of bacterial growth was due to bioactivity of either the lignin in unbleached paper or oxidized cellulose in bleached paper, there would likely be more dramatic differences in the mortality rates between the groups overtime. The higher hydrophobicity may also explain the lower extraction efficiency of unbleached samples. The more hydrophilic bleached fibers swell and loosen to a greater extent than unbleached fibers when submerged in the saline solution. The bleached fibers also lack the additional structural rigidity given by the presence of lignin. Both effects result in the disintegration of the bleached fiber network under vortex agitation. The free fibers expose more surface area to the medium and thus release nearly twice as many microbes into the saline solution.

More generally, our results show that these microbes can survive for longer than one month on paper materials at ambient conditions with very low mortality. Both bacterial species studied can form biofilms which may have enhanced their survivability in unfavorable conditions [36,37]. As previously mentioned, the survivability of *Listeria* spp. and *Salmonella* spp. (among others), on surfaces is highly strain-dependent and can vary from hours to months depending on the experimental conditions [10,15,16]. The survivability of these microbes in this study is much greater than other studies performed on similar microorganisms. As previously referenced, Sirsat et al. reported more than 1000-fold decreases in the bacterial populations on cardboard coupons after two days of incubation. The authors also reported *Listeria* spp. having much lower survivability than *Salmonella* spp. on paper [14]. Similarly, Siroli et al. observed reduction in bacterial populations greater than a 100-fold CFU/cm^2^ in 48 h after inoculation for all species and surfaces, and in some cases below the limit of detection as soon as 1 h after inoculation [11]. Differences in the bacterial strains studied as well as storage humidity, nutrient availability, and temperature likely contribute to the discrepancies in reports (see supplemental material, Section 1). For instance, using saline instead of nutrient broth for the inoculation media will result in an increase in the concentration of NaCl during evaporation, essentially dehydrating the cells. These discrepancies should be evaluated systematically in future work and a standard procedure should be developed in accordance to more realistic conditions.

The high survivability of these foodborne bacterial pathogens poses a problem in the food and agricultural industries with a high chance of cross-contamination when utilizing paper-based packaging materials [12]. Farmers, food transportation, and restaurants should be cautious when reusing and recycling these materials after exposure to dairy products or raw meat and eggs. Also, the generation of biofilms in paper recycling plants pose a threat to production operations and quality control [[38], [39], [40]].

## 5 Conclusions

Foodborne bacterial pathogens pose a notable risk to human health and may result in great economic losses in the food and agriculture industries. The results presented in this research the survivability of two model organisms for foodborne bacterial pathogens, *L. innocua* and *S.* Typhimurium, on bleached and unbleached paper surfaces. This work showed that the bacteria proliferate on both bleached and unbleached paper for 48 h after inoculation and experience very little losses over a 40-day period in the presence of sufficient nutrients. It was also presented that lignin, which has been generally thought to have antimicrobial properties, did not greatly inhibit the bacterial growth or survivability in paper materials. In fact, the lignin-containing samples (unbleached paper) exhibited higher microbial growth than that of non-lignin containing samples (bleached paper). This was attributed to the higher hydrophobicity of the fibers that may reduce the wicking of the aqueous nutrient broth, thereby improving the accessibility of nutrients. One of the limitations to this study was the limited number of organisms studied and the organisms studied were non-pathogenic strains. However, the results presented here, along with previously published studies, clearly indicate the need for the antibacterial additives for food-packaging materials. Lignin may be a potential candidate for a bio-based antimicrobial packaging additive but will likely require further modification to observe any substantial effect. The food industry has been challenged by the need for technologies that provide safe and effective microbial control. This research aids in the understanding of the complexity of studying microbial behavior on paper surfaces and similar materials.

## Author contribution statement

Jacob D. Zwilling: Conceived and designed the experiments; Performed the experiments; Analyzed and interpreted the data; Wrote the paper.

Jason Whitham: Conceived and designed the experiments; Performed the experiments; Analyzed and interpreted the data.

Franklin Zambrano; Alonzo Pifano: Analyzed and interpreted the data.

Amy Grunden; Hasan Jameel; Richard Venditti; Ronalds Gonzalez: Conceived and designed the experiments; Contributed reagents, materials, analysis tools or data.

## Funding statement

This research was supported by the National Institute of Food and Agriculture [1253354].

## Data availability statement

Data included in article/supp. material/referenced in article.

## Declaration of interest’s statement

The authors declare no competing interests.
