# Supplementary material for: Survivability of Salmonella Typhimurium (ATCC 14208) and Listeria innocua (ATCC 51742) on lignocellulosic materials for paper packaging
Source: Heliyon. 2023 Mar 9;9(3):e14122. doi: 10.1016/j.heliyon.2023.e14122 (PMC10025085; doi:10.1016/j.heliyon.2023.e14122)
Supplement: Multimedia component 1 [file mmc1.docx]

**Supplementary Material**

**Survivability of Foodborne Bacterial Pathogens on Lignocellulosic Materials for Paper Packaging**

**Jacob D. Zwilling^1^, Jason Whitham^2^, Franklin Zambrano^1^, Alonzo Pifano^1^, Amy Grunden^2^, Hasan Jameel^1^, Richard Venditti^1^, and Ronalds Gonzalez^1*^**

^1^Department of Forest Biomaterials, North Carolina State University, Biltmore Hall, Campus Box 8005, Raleigh, NC 27695, USA

^2^Department of Plant and Microbial Biology, 4550A Thomas Hall, Campus Box 7612, North Carolina State University, Raleigh, NC 27695, USA

Contents

[1. Short term effects of storage conditions 2](#_Toc91616364)

[1.1 Methods 2](#_Toc91616365)

[1.2 Results 2](#_Toc91616366)

[2. JSL code for comparison of slopes test 3](#_Toc91616367)

[3. Non-linear model of bacterial growth and decay 4](#_Toc91616368)

[4. Microscopic images of paper specimens 6](#_Toc91616369)

# Short term effects of storage conditions

## Methods

The paper specimens were stored on the laboratory benchtop in sterile, aerobic plastic petri dishes and inoculated with the bacterial inocula suspended in either TSB+ media or saline solution (NaCl, 8.5 g/L).

## Results


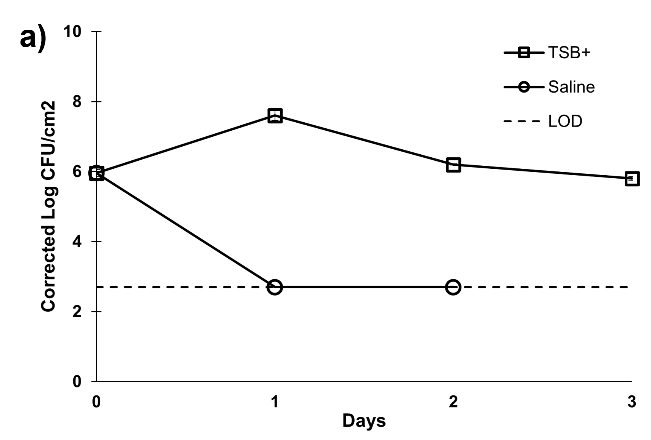

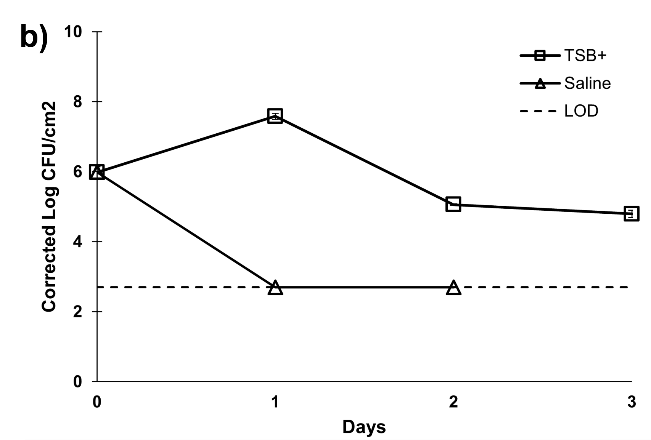


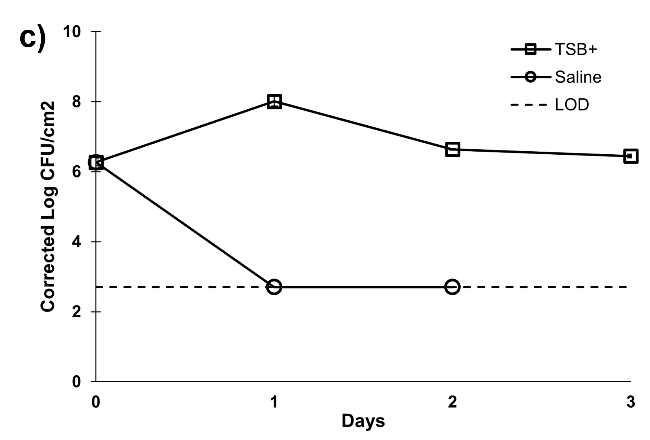

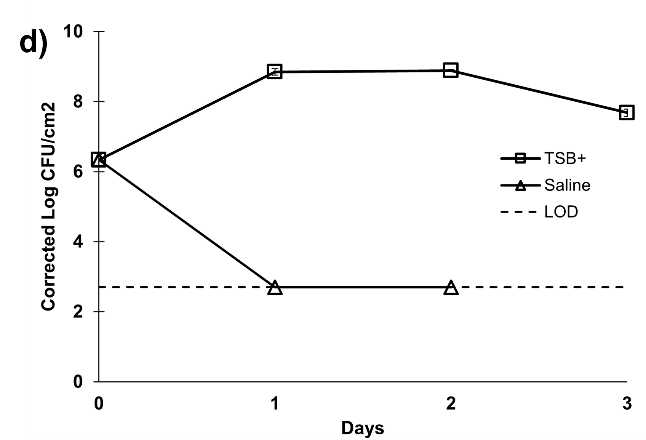


Figure S1: Short term growth and survivability of *Listeria innocua* (a,c) and *Salmonella* Typhimurium (b,d) on bleached (a,b) and unbleached (c,d) paper specimens stored in sterile petri dishes inoculated with nutrient media (TSB+) or saline solution (8.5 g/L NaCL).

# JSL code for comparison of slopes test

Fit Model(

Y( :Adjusted ),

Effects(

:BACTERIA,

:PAPER,

:BACTERIA * :PAPER,

:Day,

:BACTERIA * :Day,

:PAPER * :Day,

:BACTERIA * :PAPER * :Day

),

Personality( "Standard Least Squares" ),

Emphasis( "Minimal Report" ),

Run(

:Adjusted << {Summary of Fit( 1 ), Analysis of Variance( 1 ),

Parameter Estimates( 1 ), Scaled Estimates( 0 ),

Plot Actual by Predicted( 0 ), Plot Regression( 0 ),

Plot Residual by Predicted( 0 ), Plot Studentized Residuals( 0 ),

Plot Effect Leverage( 0 ), Plot Residual by Normal Quantiles( 0 ),

Box Cox Y Transformation( 0 ), Custom Test(

[0 0 0 0 0 0 2 2 0,

0 0 0 0 0 2 0 2 0,

0 0 0 0 0 2 2 0 0,

0 0 0 0 0 2 -2 0 0,

0 0 0 0 0 2 0 -2 0,

0 0 0 0 0 0 2 -2 0],

Label( "compare slopes" )

)}

),

SendToReport(

Dispatch(

{"Response Adjusted", "Custom Test"},

" ",

TextEditBox,

{Set Text( "compare slopes" )}

)

)

)

# Non-linear model of bacterial growth and decay


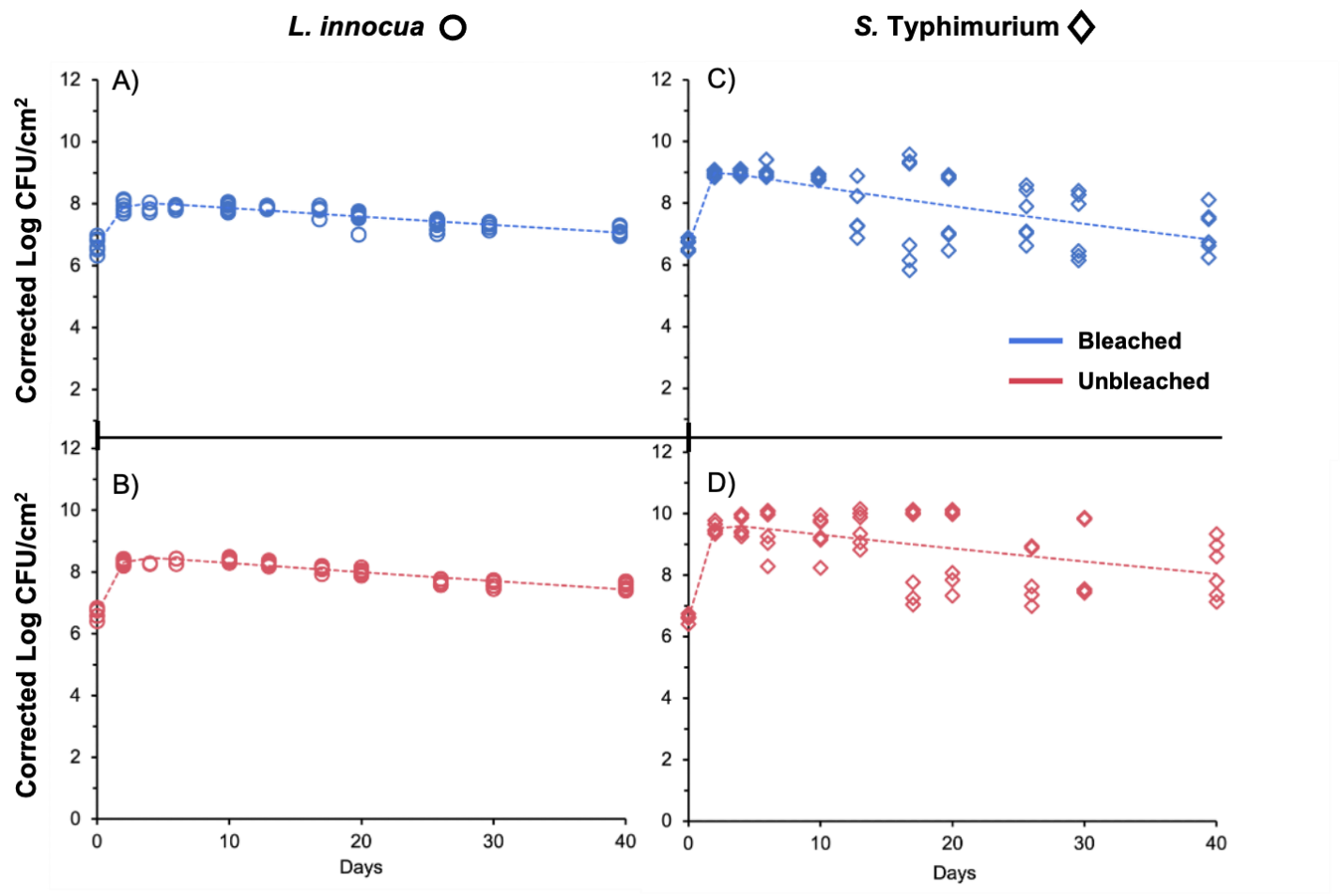


**Figure S2:** Nonlinear fit model for each of the four conditions (two bacteria, two papers). The average log CFU/m2 was corrected for the extraction efficiencies (ε) as described previously. Parameter estimates and standard errors are displayed in Table 2.

**Table S1:** Nonlinear model cell growth and mortality parameters calculated using JMP nonlinear modeling program. In Eq. S1, y_max_ is the estimated maximum bacterial count (log transformed) over the period studied, y_0_ is the estimated starting bacterial count. The model was fitted using log-transformed bacterial counts (log CFU). Growth (α) and mortality (β) are constant rate parameters. SE is the standard error of the estimated rate values.

|  | | $\frac{\boldsymbol{y}_{\boldsymbol{max}}\boldsymbol{*}\boldsymbol{y}_{\boldsymbol{0}}}{\left( \boldsymbol{y}_{\boldsymbol{max}}\boldsymbol{-}\boldsymbol{y}_{\boldsymbol{0}} \right)\boldsymbol{*}\boldsymbol{e}^{\boldsymbol{-t*}\boldsymbol{\alpha}}\boldsymbol{+}\boldsymbol{y}_{\boldsymbol{0}}\boldsymbol{*}\boldsymbol{e}^{\boldsymbol{t*}\boldsymbol{\beta}}}$ | | | (S1) | |
| --- | --- | --- | --- | --- | --- | --- |
| ***L. innocua*** | | | | | | |
| **Parameter** | **Bleached** | | **SE** | **Unbleached** | | **SE** |
| y_max_ | 8.12 | | 0.05 | 8.59 | | 0.05 |
| y_0_ | 6.69 | | 0.06 | 6.66 | | 0.06 |
| Growth (α) | 1.09 | | 0.21 | 1.18 | | 0.18 |
| Mortality (β) | 0.0036 | | 0.0003 | 0.0036 | | 0.0003 |
| ***S.* Typhimurium** | | | | | | |
| **Parameter** | **Bleached** | | **SE** | **Unbleached** | | **SE** |
| y_max_ | 9.19 | | 0.25 | 9.79 | | 0.24 |
| y_0_ | 6.64 | | 0.34 | 6.61 | | 0.34 |
| Growth (α) | 1.93 | | 2.87 | 1.65 | | 1.23 |
| Mortality (β) | 0.007 | | 0.0013 | 0.0049 | | 0.0012 |

# Microscopic images of paper specimens


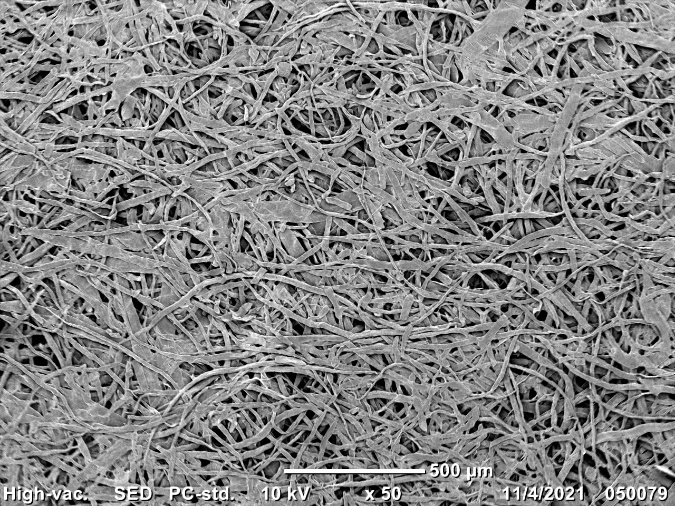

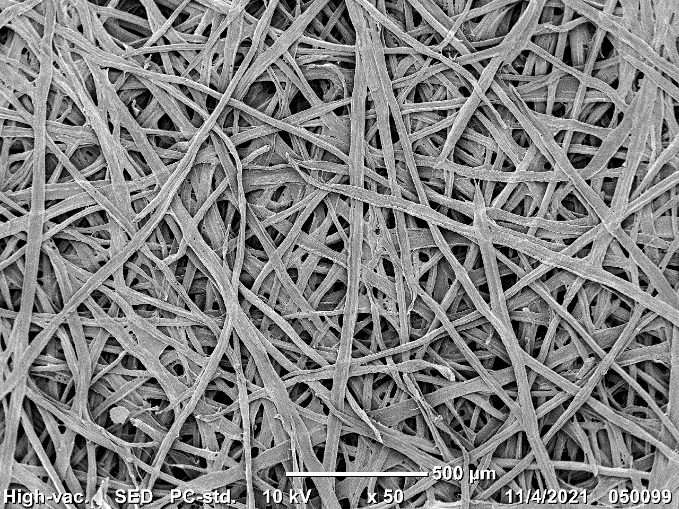


**Figure S3**: Scanning electron microscope image of bleached (left) and unbleached (right) paper specimens. SEM images were taken of the paper specimens with a JEOL JCM-6000 Benchtop SEM. The paper specimens were coated with gold using a sputter coater directly prior to image acquisition.
